# Return to Sports and Physical Activity After Total and Unicondylar Knee Arthroplasty: A Systematic Review and Meta-Analysis

**DOI:** 10.1007/s40279-015-0421-9

**Published:** 2016-01-07

**Authors:** Suzanne Witjes, Vincent Gouttebarge, P. Paul F. M. Kuijer, Rutger C. I. van Geenen, Rudolf W. Poolman, Gino M. M. J. Kerkhoffs

**Affiliations:** Department of Orthopaedic Surgery, Foundation FORCE (Foundation for Orthopaedic Research Care and Education), Amphia Hospital, Breda, The Netherlands; Department of Orthopaedic Surgery, Academic Medical Centre, ACES (Academic Centre for Evidence-based Sports medicine), ACHSS (Amsterdam Collaboration for Health and Safety in Sports), Meibergdreef 9, 1105 AZ Amsterdam, The Netherlands; Coronel Institute of Occupational Health, Academic Medical Centre, University of Amsterdam, Amsterdam, The Netherlands; Department of Orthopaedic Surgery, Onze Lieve Vrouwen Gasthuis, Amsterdam, The Netherlands

## Abstract

**Background:**

People today are living longer and want to remain active. While obesity is becoming an epidemic, the number of patients suffering from osteoarthritis (OA) is expected to grow exponentially in the coming decades. Patients with OA of the knee are progressively being restricted in their activities. Since a knee arthroplasty (KA) is a well accepted, cost-effective intervention to relieve pain, restore function and improve health-related quality of life, indications are expanding to younger and more active patients. However, evidence concerning return to sports (RTS) and physical activity (PA) after KA is sparse.

**Objectives:**

Our aim was to systematically summarise the available literature concerning the extent to which patients can RTS and be physically active after total (TKA) and unicondylar knee arthroplasty (UKA), as well as the time it takes.

**Methods:**

PRISMA guidelines were followed and our study protocol was published online at PROSPERO under registration number CRD42014009370. Based on the keywords (and synonyms of) ‘arthroplasty’, ‘sports’ and ‘recovery of function’, the databases MEDLINE, Embase and SPORTDiscus up to January 5, 2015 were searched. Articles concerning TKA or UKA patients who recovered their sporting capacity, or intended to, were included and were rated by outcomes of our interest. Methodological quality was assessed using Quality in Prognosis Studies (QUIPS) and data extraction was performed using a standardised extraction form, both conducted by two independent investigators.

**Results:**

Out of 1115 hits, 18 original studies were included. According to QUIPS, three studies had a low risk of bias. Overall RTS varied from 36 to 89 % after TKA and from 75 to >100 % after UKA. The meta-analysis revealed that participation in sports seems more likely after UKA than after TKA, with mean numbers of sports per patient postoperatively of 1.1–4.6 after UKA and 0.2–1.0 after TKA. PA level was higher after UKA than after TKA, but a trend towards lower-impact sports was shown after both TKA and UKA. Mean time to RTS after TKA and UKA was 13 and 12 weeks, respectively, concerning low-impact types of sports in more than 90 % of cases.

**Conclusions:**

Low- and higher-impact sports after both TKA and UKA are possible, but it is clear that more patients RTS (including higher-impact types of sports) after UKA than after TKA. However, the overall quality of included studies was limited, mainly because confounding factors were inadequately taken into account in most studies.

**Electronic supplementary material:**

The online version of this article (doi:10.1007/s40279-015-0421-9) contains supplementary material, which is available to authorized users.

## Key Points

**Table:** 

Return to sports is possible after knee arthroplasty, but seems more likely after unicondylar arthroplasty than after total knee arthroplasty, particularly concerning higher-impact types of sports.
In the included studies, little attention was given to possible confounding factors, such as preoperative sports level, restricting comorbidities, and negative advice from surgeons.
We recommend generalising the definition of the assessment of the preoperative sports level to the ‘presymptomatic phase’, as this plays an important role in defining return to sports percentages.

## Introduction

Patients with knee osteoarthritis (OA) are progressively restricted in their daily functioning, working and sports activities, making them less active than they would like to be. A knee arthroplasty (KA) is a well accepted, reliable and suitable surgical procedure for end-stage OA patients to relieve pain, to return to function, and to improve health-related quality of life [1]. However, literature concerning the extent to which patients can return to sports (RTS) and physical activity (PA) after both total (TKA) and unicondylar knee arthroplasty (UKA) is sparse.

People are not only living longer than before, they also want to stay active and engaged in their working activities up to and after retiring [1, 2]. According to demographic projections in the Netherlands, it is expected that the number of OA patients will increase exponentially between 2007 and 2040. Subsequently, an increase in KAs of 297 % from 2005 to 2030 is envisaged, resulting in 57,900 KAs annually in 2030 [3]. This increase is not only due to more, relatively younger patients with knee OA that want to preserve an active lifestyle without knee pain, but also to the growing burden of the obesity epidemic. For example, in the US, the demand for primary KAs is estimated to grow even more, by 673 % from 2005 to 2030, leading to 3.5 million annual procedures [4].

There is overwhelming evidence that a sedentary lifestyle is undeniably one of the most serious health problems of the 21st century [5, 6]. As a consequence, people’s wish to stay active has been stimulated by several leading international organisations that have recognised the positive effects of PA in general. International guidelines of health-enhancing PA levels have been developed and ‘exercise is medicine’ is proclaimed, by stating that PA can ameliorate affluence-related chronic diseases such as cardiovascular disease, diabetes mellitus and cancer [7]. Moreover, PA has proven to have beneficial effects on bone quality and implant fixation [8].

Since the prevalence of OA affecting the knee is rising rapidly, this disease is currently one of the leading causes of disability in adults. Due to osteoarthritic pain, physical deconditioning arises, resulting in reduced endurance for exercise, less aerobic capacity, less muscle strength, and a high risk for being overweight. Consequently, individuals with OA greatly fall short of the public health PA guidelines [9]. The possible benefits of total knee replacement in terms of pain relief and restoration of function are well documented, but impacts on health, fitness and the lower risk for coronary heart disease have also been addressed in patients who had been able to resume activities after KA [10]. Even a possible cardioprotective benefit of primary total joint arthroplasty has been described with an absolute risk reduction of 12.4 % of serious cardiovascular events after KA [11].

Furthermore, total TKA is a cost-effective medical intervention, especially concerning the younger working population suffering from OA of the knee [12, 13]. In addition to TKA, new techniques and improved implant quality of UKA have given rise to the treatment of end-stage OA of the knee. The theoretical advantages of UKA compared with TKA are that the procedure is less invasive, patients tend to achieve a better range of motion, and report a joint as feeling ‘more normal’ [14].

As a consequence of higher patient expectations regarding activities after knee replacement, clinicians are increasingly forced to question how much sports activity a patient can participate in after knee replacement, and what kind of sports activities are acceptable [15, 16]. All doctors, but especially sports medicine physicians and orthopaedic surgeons, should counsel patients regarding an active lifestyle, including when they have to undergo KA. However, synthesised data to provide reliable answers to the questions of end-stage OA patients regarding sports activities after knee replacement are lacking. Most available recommendations, such as The Knee Society consensus recommendations of 1999, are based on expert opinions from surveys rather than on evidence-based summaries of good quality research [17–21].

However, as patients are increasingly participating in a shared decision-making process, clinicians are expected to inform and advise them according to scientific knowledge rather than ‘gut feelings’. Consequently, the aim of this review is to systematically summarise the available scientific literature on our research questions. Our primary research question is ‘to what extent do patients RTS after total and unicondylar KA, and how long does this take?’. Our second research question is ‘to what extent can patients return to PA after total and unicondylar KA?’

## Methods

### Search Strategy

The PRISMA (Preferred Reporting Items for Systematic reviews and Meta-Analyses) statement was used for this systematic review [22]. A research protocol for this review was agreed by all co-authors before starting the literature searches. The study protocol was published online at the PROSPERO International prospective register of systematic reviews (http://www.crd.york.ac.uk/PROSPERO/) under registration number: CRD42014009370.

The electronic databases MEDLINE (biomedical literature) via PubMed, Embase via OvidSP, and SPORTDiscus (sports and sports medicine literature) via EBSCO were searched for relevant literature. Searches were performed up until January 5, 2015. In all three databases the following three categories of keywords (and related synonyms) were used to build a sensitive search strategy and to provide a systematic search: ‘knee arthroplasty’, ‘sports’ and ‘recovery of function’. In MEDLINE we strived to use medical subject headings (MeSH), otherwise we searched the title and/or abstract (tiab). Furthermore, search terms were truncated through the use of a * symbol in order to find all terms beginning with a specific word. Within each keywords category, the different synonyms were combined using the Boolean command OR, and categories were linked with the Boolean command AND. The exact details of the search strategy can be found in the electronic supplementary material, Appendix S1.

### Inclusion Criteria and Study Selection

The first author (SW) selected suitable studies with the assistance of a medical student and input from a medical librarian of the Academic Medical Centre (AMC). Inclusion criteria were (1) knee OA patients who underwent total and/or unicondylar KA; who (2) were active in a sport before the surgery and intended to resume or intensify their sporting activity; and (3) that included an outcome measure of interest to the authors. The primary outcome was the percentage (and number) of patients to RTS (preferably described in terms of sports level, duration and frequency) and time to RTS. Secondary outcomes were specific PA outcomes measures, namely University of California, Los Angeles (UCLA) rating score, Tegner-Lysholm rating scale and Grimby score [23–25].

The reference lists of selected articles were screened to identify additional articles to be included. We also performed a forward search using ‘Web of Science’ to see which of these papers were referred to by other papers after they had been published.

### Methodological Quality

We assessed the risk of bias of the studies using the Quality in Prognosis Studies (QUIPS) tool [26]. This quality assessment method considers six domains of potential biases: (1) study population; (2) study attribution; (3) prognostic factor information; (4) measurement of and controlling of confounding variables; (5) measurement of outcomes; and (6) analysis approaches. The first author (SW) assessed the quality of all selected articles, and this was repeated by two other authors (VG and PK), who each assessed risk of bias of 50 % of the selected articles. We customised the tool to our review by defining the issues of the domains to be scored. The details of these issues can be found in the electronic supplementary material, Appendix S2. In domain 2, we adjusted for a minimum follow-up period of 1 year, according to the literature, which states that the greater part of the knee function will have been regained at 1 year after surgery [27–29].

By assessing response rate and information about non-responders, we chose a cut-off point of 20 %, based on previous studies in this field [30, 31]. In domain 5, concerning study confounding, we identified confounding variables for activity from previous research we found on this subject before performing this systematic review [1, 28, 32–35]. We rated the issues per domain separately as ‘yes’, ‘no’, ‘partial’ or ‘unsure’, which then led to a risk of bias for each domain to being ‘low’, ‘moderate’ or ‘high’. We considered a study to have an overall low risk of bias when the methodological risk of bias was rated as low or moderate in all six domains, with at least four domains being rated ‘low’. A study was rated as having an overall high risk of bias if two or more of the domains scored ‘high’. In-between quality was scored as ‘moderate’.

### Data Extraction

The first author (SW) extracted data from all selected original articles, and this was repeated by two other authors (VG and PK), each extracting data from 50 % of the included articles.

All authors used a standardised data extraction form including the following topics: (1) study information: author, year, country and reference number; (2) study design and follow-up; (3) information about study population: cohort, population size, sex, age, body mass index (BMI), comorbidities, and type of OA (primary or secondary causes, such as systematic inflammatory disease or post-traumatic arthritis); (4) description of rehabilitation protocols used; (5) definition of the outcome measures; (6) preoperative activity and definition (e.g. presymptomatic or at time of surgery); (7) postoperative activity; (8) RTS percentages and time to RTS; and (9) confounding factors taken into account for RTS, such as sex, BMI, restricting comorbidities, complications, preoperative sports level, surgeon recommendations or other psychosocial influencing factors.

### Pooling Data

From the studies that described pre- and/or postoperative participation in specific types of sports and/or times to RTS, data were pooled and categorised into low-, intermediate- or high-impact sports, according to the levels of impact on the knee joint (see electronic supplementary material, Appendix S3). This classification is in compliance with Vail and Mallon [36] and supported by a biomechanical study from Kuster et al. [37], in which both peak loads and flexion angles of the knee were considered. We calculated pooled RTS percentages by comparing pooled pre- and postoperative sports participation data.

## Results

### Literature Search

**Fig. 1:**
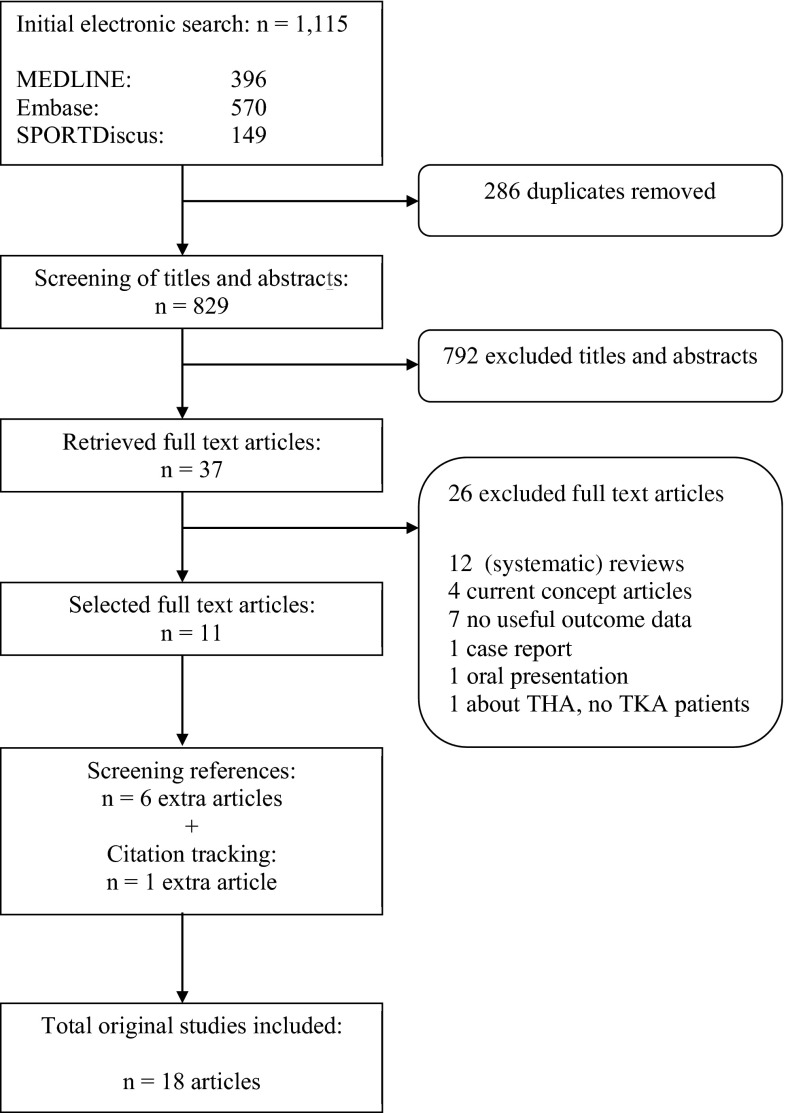
PRISMA flowchart of search strategy. *THA* total hip arthroplasty, *TKA* total knee arthroplasty

We retrieved a total of 1115 potentially relevant citations from our search. After deleting 286 duplicates and applying the inclusion criteria to titles and abstracts, we reviewed 37 full-text articles, 12 of which were review articles. We excluded these from data extraction, as with 14 other articles, which were excluded for various reasons such as current concept reviews, case reports or studies not presenting outcomes of our interest. On both reviews and the included 11 articles, we performed reference screening and forward citation tracking, which resulted in seven additional articles being included. The article by Jahromi et al. [38] was excluded because the same UKA cohort was described in the article by Walton et al. [39]. Finally, 18 original studies were included. The PRISMA flowchart of our search procedure can be found in Fig. 1.

### Included Studies

All of the included studies were observational, 13 being cross-sectional studies, three prospective studies and two retrospective cohort studies. Two studies were performed in Australia [39, 40], one in Austria [41], two in France [42, 43], four in Germany [44–47], one in Italy [48], one in Korea [49], one in Switzerland [14], four in the UK [50–53] and two in the US [54, 55]. From three of the 18 included articles, data about sports activities after both TKA and UKA was able to be extracted, from ten after TKA and from five after UKA, of which one article specifically described outcomes after lateral UKA. Of the 13 articles with respect to data about RTS after TKA, the study population of eight studies was a non-selected group of KA patients. Five studies examined a selected population of patients. Two of these studies examined patients younger than 75 years old, one study assessed patients younger than 65 years old, another study concerned non-revised patients younger than 55 years old and the last study evaluated licensed judokas (i.e. people who participate in judo, which is a method of defending oneself or fighting without the use of weapons, based on jujitsu) with black belts of 60 years and older.

This latter study described outcomes of two different age groups, namely patients younger than 55 years and patients aged 65–75 years. Of the eight articles with respect to data about RTS after UKA, seven studies examined a non-selected cohort and one study examined a cohort of patients younger than 75 years old.

The total number of patients in the 13 TKA cohorts was 3261 and the mean age of these patients varied between 49 and 73 years at time of surgery, with ranges from 21 to 96 years. Mean BMI varied from 27 to 34 kg/m^2^ with ranges from 16 to 44 kg/m^2^, but was clearly described in three of the 13 included studies. Only three of the 13 studies provided information concerning possible restricting comorbidities on levels of PA. Five of the 13 studies provided information about the rehabilitation protocols followed.

The total number of patients in the eight UKA cohorts was 662. The mean age at time of surgery of these patients varied between 59 and 72 years, with ranges from 21 to 95 years.

The BMI of the patients was specified in three of eight UKA cohorts and was described as means of the total cohort of 26 and 28 kg/m^2^ (range 20–42) and Pietschmann et al. [46] described a mean BMI in active patients of 28 kg/m^2^ (range 20–56) and in inactive patients of 29 kg/m^2^ (range 19–43). Fisher et al. [51] took into account medical problems restricting PA after UKA and four of eight studies provided information about the rehabilitation protocols. The results of the data extraction are presented in Table 1 for articles concerning data of RTS after TKA and in Table 2 for articles concerning data of RTS after UKA.

**Table 1:** Return to sports after TKA: data extracted from studies included in the review (*N* = 13)

Study	Study design	Study population	Rehabilitation protocol	Outcome measures	Preoperative (pre) activity + definition	Postoperative (post) activity	RTS + time to RTS	Confounding factors
Argenson et al. (2008) France [42]	Prospective Follow-up: 3 y (range 2–4)	Non-selected 455 pts, 516 KAs 146 (33 %) M 299 (67 %) F Mean age: 71.6 y (range 22–96) Mean BMI: 28.3 kg/m^2^ (range 16–44) Co: ? OA: 92 % primary, 2 % RA, 6 % other	Full weight bearing	General (*n* = 412)	Regarding sports: Inactive: 34 % Limited in ADL: 54 % Active in work/sports: 9 % Unknown: 3 %	Sports level: =14 % >72 % <14 % Mainly walking, hiking, swimming, exercising, cycling, golfing	86 % Time to RTS: 6 mo (±4)	Age Sex BMI Pre sports level Complications Expectations Partly mentioned, not adjusted for
				UCLA (*n* = 412)	Unknown Pre-sports activity level at time of surgery	6.9 (±1.6)		
Bock et al. (2003) Austria [41]	Cross-sectional Follow-up: 74 mo (24–156) Language: German	Selected, <65 y 138 pts, 184 KAs 28 (20.3 %) M 110 (79.7 %) F Mean age: 55.3 y (21–65) Mean BMI: ? Co: 7 pts (abcess, spondylodiscitis, sciatica, CVA, kidney, cardiopulmonary) OA: Primary 63 % Polyarthritis 28 % Post-traumatic 4 % Inflammatory 1.4 % Other 3.4 %	Uncemented: 3-point gait during 6 wks Cemented: 4-point gait pattern 6 wks After 6 wks: similar stepwise mobilisation for every pt	General active (*n* = 138) Sports participation:	*n* = 111 (80.4 %)	*n* = 104 (75.4 %)	*n* = 99 (89 %) RTS%	Age Co-morbidities Pre sports level Mentioned in results or discussion, not adjusted for
				- Walking	99	99 + 4 = 103	100	
				- Cycling	47	20	42.6	
				- Swimming	43	37 + 1 = 38	86.0	
				- Hiking	28	18	64.3	
				- Skiing	7	1	14.3	
				- Langlauf	4	2	50	
				- Mountain climbing	4	0	0	
				- Tennis	2	0	0	
				- soccer	3	0	0	
				- Stationary biking	0	7	?	
				- Aquajogging	0	1	?	
				General: 12 pts stopped post, 5 pts restarted sports (4 × walking, 1 × swimming)		Active yes/no		
				UCLA	Unknown	5.9/2.9		
				Tegner	Unknown	3.9/1.8		
					No information concerning definition pre sports		Time to RTS: 4.7 mo (3–15)	
Bradbury et al. (1998) UK [50]	Cross-sectional Follow-up: 5 y (3–7)	Non-selected 160 pts, 208 KAs [RTS unilateral 61 %, bilateral (simultaneous TKA) 75 %] 30 (8 %) M 339 (92 %) F Mean age: 68 y (27–87) Mean BMI: ? Co: 55 co-morbidities that limited their activity (pre) → no RTS OA: 142/159 (89.3 %) primary	Early post-op mobilisation and full weight bearing was permitted as tolerated with supervision of physical therapist	General active (*n* = 160)	*n* = 79 (49.4 %)	*n* = 51 (31.9 %)	65 %	Co-morbidities − Pre sports level + Complications − Psychosocial (motivation) + Surgeon advice: all pts were advised that high-impact sports were not recommended and low-impact sports were acceptable
				In last year before surgery	56*	43/56 (77 %)		
				Sports participation:			RTS%	
				- Golf	51	39	76.5	
				- Bowls	32	29	91	
				- Tennis	30	6	20	
				- Cycling	1	4	>100	
				- Croquet	4	4	100	
				- Golf + cart	3	3	100	
				- Cricket	3	2	66.7	
				- Body surf	1	1	100	
				- Bush walk	2	1	50	
				- Tai chi	0	1	?	
				- Rugby, skiing, football, running	1	0	0 %	
					No information concerning definition pre sports			
Chang et al. (2014) Korea [49]	Retrospective survey Follow-up: 2 y (1–3)	Non-selected 369 pts 30 (8 %) M 339 (92 %) F Mean age: 68.8 y (50–83) Mean BMI: 27.4 kg/m^2^ (19.3–39.1) Co: 50 (14 %) medical limitation OA: 331 (95 %) primary	No description	Regular^a^ activity (*n* = 369)	71 %	76 %	+5 %*	Sex Age BMI Pre sports level Recall bias All suggested as possible confounders and ANCOVA of age, BMI and preop level, but results not presented
				Sports participation:			RTS%	
				- Walking	177	221	>100	
				- Swimming	79	85	>100	
				- Bicycling	60	80	>100	
				- Hiking	34	22	65	
				- Gymnastics	14	17	>100	
				- Badminton	9	6	67	
				- Running	7	5	74	
				- Golf	7	2	26	
				- Table tennis	5	3	57	
				- Gateball	3	4	>100	
				UCLA mean subscore	4.5 *N* (%)	4.8 *N* (%)	∆ 0.3*	
				UCLA <4	90 (24)	39 (11)		
				UCLA 4–6	240 (65)	296 (80)		
				UCLA >6	36 (11)	34 (9)		
				VAS satisfaction in general (post)		Active yes/no 7.9/7.2	∆ 0.7^#^	
				VAS satisfaction about activity (post)		7.5/6.3	∆ 1.2^#^	
					No information concerning definition pre sports			
Chatterji et al. (2005) Australia [40]	Cross-sectional survey Follow-up: ? ‘between 1 and 2 y post’	Non-selected 144 pts 64 (44 %) M 80 (56 %) F Mean age: 70.8 y (SD 10.4) Mean BMI: ? Co: ? OA: 136 (94 %) primary	Post-PT was carried out in the hospital until discharge, then performed their own rehabilitation, as instructed prior to discharge Only occasionally was formal out-pt PT needed (no further details provided)	1 or more sports (*n* = 144) Sports participation:	*n* = 122 (85%)	*n* = 108 (75 %)	81 % Same sport 69 % RTS%/time wks	Sex: M > F Men were significantly more sport oriented than women, both pre- and postoperative
				- Volleyball	0	1	?/−	
				- Squash	1	0	0/−	
				- Croquet	2	1	50/5	
				- Badminton	1	1	100/6	
				- Rowing	3	0	0/−	
				- Snow skiing	1	0	0/−	
				- Hiking	2	0	0/−	
				- Jogging	4	0	0/−	
				- Aqua aerobics	8	12	>100/6.9	
				- Bush walking	8	3	38/8	
				- Tennis	14	2	14/30	
				- Fishing	18	12	67/26	
				- Cycling	10	5	50/12.5	
				- Exercise classes	12	9	75/12	
				- Bowling	22	17	77/18.3	
				- Golf	19	9	47/13	
				- Swimming	27	22	81/13.1	
				- Exercise walking	94	104	>100/8.7	
				Grimby score	Unknown	2.8		
				Mean sport score (n. of sports)			N. of sports: −20 % −12 % ∆ 0.7*	
				- Overall	1.76	1.41		
				- Sporting patients	2.1	1.8		
				- M	2.4	1.7		
				- F	1.4	1.1		
				Perceived effect of TKA in sports function (*n* = 106) (meaning: 1 = improved to 10 = stopped completely)		All 4.3 (SD 3.4) M 4.2 (SD 3.3) F 4.7 (SD 3.6)		
					No information concerning definition pre sports			
Diduch et al. (1997) USA [54]	Cross-sectional Follow-up: 8 y (3–18)	Selected, unrevised, <55 y (post-traumatic) OA 84 pts, 103 KAs 29 (35 %) M 55 (65 %) F Mean age: 51 y (22–55) Mean BMI: ? Co: ? OA: 64 % primary, 36 % post-traumatic	No description	Sports participation		*N* (%) of *n* = 80		No information Only comment concerning usual cautions to avoid activities, but not adjusted for
				- Walking (3.2 km)		48 (60)		
				- Cycling		42 (53)		
				- Golf		19 (24)		
				- Treadmill running		16 (20)		
				- Aerobics		7 (8.8)		
				- Tennis		9 (11.3)		
				- Stairclimbing machine		10 (12.5)		
				- Hiking		13 (16.3)		
				- Downhill skiing		6 (7.5)		
				Tegner	1.3 (0–4)	3.5 (1–6)	>97.5 % =2.5 %	
				Tegner >5 [meaning: regular participation in tennis, downhill skiing, cycling or strenuous (farm or construction) work)		19 (24 %)		
					No information concerning definition pre sports			
Hopper and Leach (2008) UK [52]	Cross-sectional Follow-up: 21.6 mo (13–42)	Selected, ≤75 y *n*: 76 pts 34 (45 %) M 42 (55 %) F Mean age: 62.1 y (35–75) Mean BMI: ? Co: ? OA: ?	No description	Low-impact sports (*n* = 76) Sports participation:	*n* = 55	*n* = 36	64 %^c^ RTS%	Complications (pain 42.9 %, instability 25.7 %) Co-morbidities Both mentioned but not adjusted for
				- Swimming	30	23	76.7	
				- Bowls	17	7	41.2	
				- Golf	17	5	29.4	
				- Dancing	16	11	68.8	
				- Cycling	15	7	46.7	
				VAS satisfaction (about activities):		%	Time to RTS: 4.1 mo	
				- Very satisfied		56		
				- Satisfied		25		
				- Fairly satisfied		12		
				- Dissatisfied		8		
				Sports frequency (*N* per week)	3.0	2.0	∆ −1.0*	
					No information concerning definition pre sports			
Huch et al. (2005) Germany [44]	Longitudinal, multicentre prospective trial Follow-up: 5 y	Selected, <75 y 389 pts 106 (27.3 %) M 283 (72.7 %) F Mean age: 66.3 y (SD 6.4) Mean BMI: ? (no mean, >75 % overweight) Co: ? OA: ?	No description	General (*n* = 389)	94 % during life	34 %	36 %	List of reasons for reduction: Complications (pain 16.3 %) Co-morbidities (pain elsewhere 29.2 %) Precaution 41.7 % (pts advised against high-impact) Multivariable regression: Age: − Sex: M > F Pre sports level: +
					42 % at time of surgery (pre)		81 %	
				Sports participation:	*n* = during life/pre		RTS%	
				- Cycling	55/27	32	58	
				- Hiking	38/2	28	73.7	
				- Swimming	45/27	36	80	
				- Downhill skiing	20/2	2	10	
				- Gymnastics	24/6	8	33.3	
				- Cross-country skiing, jogging	16/2	3	18.8	
				- Tennis	6/1	2	33.3	
				- Dancing	5/1	4	80	
					Clear definition of pre sports			
Keeney et al. (2014) USA [55]	Retrospective Follow-up (<55 y): 36 mo (12–118) Follow-up (65–75 y): 31 mo (12–110)	Non-selected 412 pts, 495 KAs, divided into 2 age groups: ≤55 y *n* = 150, 181 KAs 65–75 y *n* = 262, 181 KAs Mean age: ≤55: 49 y 65–75: 69.9 y Sex: ? Mean BMI: ≤55: 34 kg/m^2^ 65–75: 32 kg/m^2^ OA: 79–95 % primary Co: ?	No description	UCLA:			RTS%	Age: no Sex: M > F BMI − Pre sports level only mentioned as possible confounder
				Group ≤55 y	3.4	4.6	10	
				Increase		57 %		
				Decrease		14 %		
				Group 65–75 y	3.8	4.9	12	
				Increase		65 %		
				Decrease		14 %		
				UCLA	>5			
				Group <55 y	13 %			
				Group 65–75 y	23 % (Age ∆*)			
					No information concerning definition pre sports			
Lefevre et al. 2013 France [43]	Cross-sectional Follow-up: 7 ± 5.2 y	Selected, judokas (licensed at French Judo federation) with black belt >60 y 8 pts, 10 KAs Sex: ? Mean age: 72.8 y (range ± 5.2) Mean BMI: ? Co: ? OA: ?	No description	Sports participation			RTS% 63 Time to RTS: 5.2 (±2.2) mo	Pre sports level mentioned as possible confounder Surgeon advice: ‘many pts RTS, despite the surgeon’s recommendations to the contrary’
				- Judo	8	5^b^		
				Other sports				
				- Walking	Unknown	7		
				- Swimming	Unknown	7		
				- Bicycling	Unknown	5		
				- Golf	Unknown	1		
					Pre sports was defined as ‘during life’			
Münnich et al. (2003) Germany [45]	Prospective Follow-up: 7 y (2–18) Language: German	Non-selected 40 pts, 42 KAs 13 (32.5 %) M 27 (67.5 %) F Mean age: 68.8 y (50–83) Mean BMI: ? Co: ? OA: ?	Only comment that possible loading on prosthesis should be taken into account during rehabilitation	Sports participation:		1 y/2 y	RTS%	Comment regarding importance of counselling by surgeon
				- Walking	28	32/30	>100	
				- Swimming	16	12/7	75	
				- Gymnastics	11	23/19	>100	
				- Cycling	16	15/15	93.8	
					No information concerning definition pre sports			
Walton et al. (2006) Australia [39]	Cross-sectional Follow-up: minimum 12 mo	Non-selected 120 pts, 142 KAs 59 (49.2 %) M 61 (50.8 %) F Mean age: 71.5 y (29–95) Mean BMI: ? Co: ? OA: 100 % primary	All pts had undergone standardised rehabilitation regimens	Sports participation		total/new	RTS%/time to RTS (wks)	Negative advice All pts were advised to cease high-impact activities such as jogging Health status mentioned as possible confounder
				- Walking	81	76/9	82.7/9.3	
				- Swimming	22	14/0	63.6/13.5	
				- Golf	15	6/0	40/12	
				- Crown green bowls	17	13/1	70.6/17.8	
				- Cycling	9	5/1	44.4/12.5	
				- Hiking	8	1/0	12.5/8	
				- Fishing	14	8/1	50/32	
				- Tennis	11	2/0	18.2/30	
				- Gymnastics	9	7/3	44.4/12.8	
						>14 % =30 % <43 % no sports 13 %		
				Grimby score		2.76 (1–6)		
					No information concerning definition pre sports			
Wylde et al. (2008) UK [53]	Cross-sectional Follow-up: ? (at least 1 y postoperative)	Non-selected 866 pts 355 (41 %) M 511 (59 %) F Mean age: 69.6 y (26–93) Mean BMI: ? Co: ? OA: ?	No description	General active in sports (*n* = 866)	*n* = 253 Pre sports defined as ‘in 3 y before surgery’	*n* = 185	RTS% 73	Reasons not to RTS: - Pain - Functional problems - Medical advice - Fear of damaging joint - Lack of confidence Age: no Sex: M > F

*ANCOVA* analysis of covariance, *BMI* body mass index, *Co* (possible restricting) co-morbidities, *CVA* cerebrovascular accident, *F* female, *KAs* knee arthroplasties, *M* male, *no* no influence, *OA* osteoarthritis, *p* probability, *post* postoperative, *pre* preoperative, *PT* physiotherapy, *pt* patient, *RA* rheumatoid arthritis, *RTS* return to sports, *SD* standard deviation, *TKA* total knee arthroplasty, *UCLA* University of California Los Angeles activity score, *VAS* visual analogue scale, *wks* weeks, *mo* months, *y* years, ? unknown or unclear, ∆ difference, * p < 0.05, ^#^ no statistical significance, = same level, > higher level or more than, < lower level or less than, – negative influence, + positive influence

^a^Not otherwise specified

^b^Hence all changed their way of practising judo

^c^80 % returned to the same or to a higher level of sports

**Table 2:** Return to sports after UKA: data extracted from studies included in the review (*n* = 8)

Study	Study design	Study population	Rehabilitation protocol	Outcome measures	Preoperative (pre) activity + definition	Postoperative (post) activity	RTS + time to RTS	Confounding factors
Fisher et al. (2006) UK [51]	Cross-sectional Follow-up: 18 mo (4–46)	Non-selected 66 pts 32 (48 %) M, 34 (52 %) F Mean age: 64 y (49–81) Mean BMI: ? Co: 4 pts restricted in PA due to medical problems OA: 100 % isolated medial compartment OA	No description	Regular sports participation (at least 1 × per month)	*n* = 42 presympt *n* = 15 <3 mo pre	*n* = 39	93 %	Age Co-morbidities Complications (revision to TKA) Described as possible confounders and taken into account in results
				Sports participation			%RTS	
				- Swimming	13	12	92.3	
				- Golf	10	10	100	
				- Dancing	6	5	83.3	
				- Bowls	3	3	100	
				- Cycling	4	3	75	
				- Hiking	3	3	100	
				- Jogging	1	1	100	
				- Gym	1	1	100	
				- Squash	1	1	100	
				UCLA	4.2 (3–6)	6.5 (3–9)	∆ 2.3*	
					Clear definition of pre sports			
Hopper and Leach (2008) UK [52]	Cross-sectional Follow-up: 22.3 mo (12–44)	Selected, ≤75 y 34 pts 20 (59 %) M 14 (41 %) F Mean age: 61.3 y (43–75) Mean BMI: ? Co: ? OA: ?	No description	Low impact sports (*n* = 34)	*n* = 30	*n* = 29	96.7 %^a^	Complications (pain 24,1 %, instability 0 %) Co-morbidities Both mentioned but not taken into account
				Sports participation			RTS%	
				- Swimming	12	9	75	
				- Bowls	14	11	78.6	
				- Golf	13	13	100	
				- Dancing	5	5	100	
				- Cycling	6	5	83.3	
				VAS satisfaction (about activities)		%		
				- Very satisfied		81		
				- Satisfied		7		
				- Fairly satisfied		7		
				- Dissatisfied		5		
				Sports frequency (N per week)	3.2	3.4	∆ +0.2^#^	
					No information concerning definition pre sports		Time to RTS: 3.6 mo	
Lo Presti et al. (2011) Italy [48]	Cross-sectional Follow-up: 24 mo (12–48)	Non-selected 53 pts 15 (28.3 %) M 38 (71.7 %) F Mean age: 59 y (46–66) Mean BMI: ? Co: ? OA: ?	Compulsory postoperative rehabilitation with restoration of muscular control was important for optimum function and return to activity (but no information about which pts followed such rehabilitation)	General performing a type of sports (*n* = 53)	*n* = 46	*n* = 49	>100 %	No information Probably low because very high % RTS
				Sports participation				
				- Cycling		14		
				- Swimming		12		
				- Dancing		6		
				- Jogging		5		
				- Tennis		5		
				- Football		5		
				- Mountain climbing		5		
				- Skiing		3		
					No information concerning definition pre sports			
Naal et al. (2007) Switzerland [56]	Cross-sectional Follow-up: 18 mo (12–28)	Non-selected 83 pts 45 (54.2 %) M 38 (45.8 %) F Mean age: 65.5 y (47–83) Mean BMI: 28.3 kg/m^2^ (19.6–39.2) Co: ? OA: 86.7 % primary 13.3 % osteonecrosis	Pts were advised not to RTS before a sufficient muscular restoration of the quadriceps and hamstrings was reached	General active in ≥1 sport (*n* = 83)	*n* = 77	*n* = 73^b^	94.8 %	Correlations: Age: no Sex: no BMI: − (weak) Pre sports: +
				Sports participation			RTS%	
				- Hiking	56	43	76.8*	
				- Cycling	49	42	85.7	
				- Downhill skiing	48	18	37.5*	
				- Swimming	42	34	81*	
				- Exercise walking	33	28	84.9	
				- Cross-country skiing	21	7	33.3*	
				- Dancing	21	6	28.6*	
				- Tennis	21	3	14.5*	
				- Jogging	18	2	11.1*	
				- Fitness	12	16	>100	
				- Mountain climbing	12	1	8.3*	
				- Soccer	10	2	20*	
				- Aerobics	6	5	83.3	
				- Hand-/volley-/basketball	6	3	50	
				- Golf	5	8	>100	
				- Gymnastics	5	6	>100	
				- Riding	4	1	25	
				- Nordic walking	3	7	>100	
				- Inline skating	3	0	0	
				- Fishing	2	2	100	
				- Ice hockey	2	1	50	
				- Snowboard	2	0	0	
				- Athletics	2	0	0	
				- Shooting	1	1	100	
				- Boxing	1	0	0	
				- Water skiing	1	0	0	
				- Home trainer	0	3	?	
				- Ice skating	0	1	?	
					Preoperative = before onset of restricting symptoms		Time to RTS (mo) <3: 46 % <6: 69 % >6: 31 %	
Pietschmann et al. (2013) Germany [46]	Cross-sectional Follow-up: 4.2 y (1–10 y)	Non-selected 131 pts 57 (44 %) M, 74 (56 %) F Mean age: 65.3 y (44–90) BMI, kg/m^2^ (mean): - Active 27.6 (20–56) - Inactive 29.3 (19–43) Co: ? OA: ?	Pts were encouraged to use crutches until completion of wound healing, but mobilise freely from the first postoperative day	General active in some type of sports	*n* = 78	*n* = 63	80.8 %	Age: no BMI: − Complications and co-morbidities were equally distributed between active and inactive pts
				Sports participation			RTS%	
				- Cycling	45	44	97.8	
				- Swimming	17	14	82.4	
				- Fitness	9	10	>100	
				- Hiking	13	13	100	
				- Alpine climbing	8	3	38.5	
				- Golf	3	3	100	
				- Gymnastics	14	12	85.7	
				- Alpine skiing	17	7	41.2	
				- Cross-country skiing	2	2	100	
				- Soccer	4	0	0	
				- Tennis	3	0	0	
				- Table tennis	1	1	100	
				- (Nordic) walking	4	10	>100	
					No information concerning definition pre sports			
Walker et al. (2014) Germany [47]	Cross-sectional Follow-up: 3 y (2.0–4.3)	Non-selected 45 pts (lateral UKA) 19 (42 %) M 26 (58 %) F Mean age: 60.1 y (36–81) BMI, kg/m^2^ (mean): - Young^c^ 26 (21–42) - Old^c^ 28 (22–36) Co: ? OA: ?	No description	General active in ≥1 sports (*n* = 45)	*n* = 42 pre = before onset of restrictive symptoms	*n* = 41	97.6 % >67 % =24 % <9 %	Reasons for changing activities: - Precaution 56 % - Less Motivation 20 % - Instability 11 % - Pain 9 % - Overweight 4 % - Restricted range of motion 2 % - Lower back pain 2 %
				Sports participation			RTS%	
				- Biking	27	33	> 100	
				- Hiking	22	18	81.8	
				- Long walks	22	20	91	
				- Nordic walking	11	11	100	
				- Fitness	9	17	>100	
				- Swimming	9	15	>100	
				- Downhill skiing	8	0	0	
				- Jogging	6	3	50*	
				- Soccer	6	0	0	
				- Tennis	5	0	0*	
				- Aquarobics	4	8	>100*	
				- Cross-country skiing	4	1	25	
				- Aerobics	4	9	>100	
				UCLA	5.3 (±2.3)	6.7 (±1.5) 2/3 > 7	Time to RTS (months) <3: 56 % <6: 78 % >6: 22 %	
				Tegner	2.9 (±1.6)	3.5 (±0.8)		
					Clear definition of pre sports			
Walton et al. (2006) Australia [47]	Cross-sectional Follow-up: ≥12 mo	Non-selected 150 pts, 183 KAs 76 (51 %) M 74 (49 %) F Mean age: 71.5 y (36–92) Mean BMI: ? Co: ? OA: 100 % primary	All pts had undergone standardised rehabilitation regimens	Sports participation		Total/new	RTS%/time RTS (wks)	Negative advice All pts were advised to cease high-impact activities such as jogging Health status mentioned as possible confounder
				*-* Walking	77	88/18	90.9/7.9	
				- Swimming	23	27/5	65.6/9.4	
				- Golf	21	15/1	66.7/13	
				- Crown green bowls	20	19/3	80/24.6	
				- Cycling	19	20/4	84.2/11.8	
				- Hiking	18	10/1	50/18.7	
				- Fishing	11	10/1	81.8/16.2	
				- Tennis	8	3/0	37.5/10	
				- Gymnastics	7	8/5	42.9/11.7	
						>13 % =54 % <19 % No sports 14 %		
				Grimby score	?	3.89 (1–6)		
					No information concerning definition pre sports			
Wylde et al. (2008) UK [53]	Cross-sectional Follow-up: ? (at least 1 y postoperative)	Non-selected 100 pts 48 (48 %) M 52 (52 %) F Mean age: 66.0 y (45–88) Mean BMI: ? Co: ? OA: ?	No description	General active in sports (*n* = 100)	*n* = 36 Pre sports defined as ‘in 3 y before surgery’	*n* = 27	75 %	Reasons not to RTS - Pain - Functional problems - Medical advice - Fear of damaging joint - Lack of confidence Age: no Sex: M > F

*BMI* body mass index, *Co* (possible restricting) co-morbidities, *F* female, *KAs* knee arthroplasties, *M* male, *no* no influence, *OA* osteoarthritis, *p* probability, *PA* physical activity, *post* postoperative, *pre* preoperative, *pt* patient, *RTS* return to sports, *TKA* total knee arthroplasty, *UCLA* University of California Los Angeles activity score, *UKA* unicondylar knee arthroplasty, *VAS* visual analogue scale, *wks* weeks, *mo* months, *y* years, ? unknown or unclear, ∆ difference, * p < 0.05, ^#^ no statistical significance, = same level, > higher level or more than, < lower level or less than, − negative influence, + positive influence

^a^88 % returned to the same or to a higher level of sports

^b^None of pre inactive pts took up new activities

^c^Median age 62 years as cut-off value

### Methodological Quality

Three of the 18 studies, namely Bradbury et al. [50], Huch et al. [44] and Naal et al. [14], were rated as having a low risk of bias, nine were scored as moderate [40, 41, 45–47, 49, 51, 52, 54] and six as high [42, 43, 48, 55]. It was notable that most studies provided no information about possible confounding factors, as six studies scored ‘high’ and eight studies ‘moderate’. The lowest risk of bias was found for the prognostic factor in which the type of prosthesis was described. No study scored a ‘high’ risk of bias in that domain. A summary of all scored risks of bias per domain is listed in Table 3.

**Table 3:** Methodological assessment according to six domains of potential biases (QUIPS)

Study (*n* = 18)	Study participation	Study attrition (follow-up)	Prognostic factor	Outcome	Confounding factor	Analysis	Overall risk of bias^a^
Argenson et al. (2008) [42]	Moderate	High	Low	Moderate	High	Moderate	High
Bock et al. (2003) [41]	Low	Moderate	Low	Moderate	Low	Moderate	Moderate
Bradbury et al. (1998) [50]	Moderate	Low	Low	Low	Low	Low	Low
Chang et al. (2014) [49]	Moderate	Moderate	Moderate	Moderate	Moderate	Low	Moderate
Chatterji et al. (2005) [40]	Moderate	Moderate	Moderate	Moderate	Moderate	Low	Moderate
Diduch et al. (1997) [54]	Moderate	Low	Moderate	Moderate	Moderate	High	Moderate
Fisher et al. (2006) [51]	Moderate	Moderate	Low	Moderate	Moderate	Moderate	Moderate
Hopper and Leach (2008) [52]	Low	Moderate	Low	Moderate	Moderate	Moderate	Moderate
Huch et al. (2005) [44]	Low	Moderate	Low	Low	Moderate	Low	Low
Keeney et al. (2014) [55]	Moderate	High	Moderate	Moderate	High	Moderate	High
Lefevre et al. (2013) [43]	Moderate	High	Moderate	Low	High	Moderate	High
Lo Presti et al. (2011) [48]	High	Moderate	Low	High	High	High	High
Münnich et al. (2003) [45]	Moderate	Moderate	Moderate	Moderate	High	Moderate	Moderate
Naal et al. (2007) [56]	Moderate	Low	Low	Low	Low	Low	Low
Pietschmann et al. (2013) [46]	Moderate	Moderate	Low	Moderate	Moderate	Moderate	Moderate
Walker et al. (2014) [47]	Moderate	Low	Low	Moderate	Low	Moderate	Moderate
Walton et al. (2006) [39]	Moderate	Moderate	Moderate	High	Moderate	High	High
Wylde et al. (2008) [53]	Moderate	High	Moderate	Moderate	High	High	High

*QUIPS* Quality in Prognosis Studies

^a^We considered a study to be of low risk of bias when the methodological risk of bias was rated as low or moderate on all of the six domains, with at least four rated as low. A study was overall scored as high risk of bias if two or more of the domains were scored as high

### Return to Sports (RTS)

Eight of the 13 studies reported data about the percentages of patients who RTS after TKA. Mean percentages of RTS varied from 36 to 89 %. Huch et al. [44] described two possible percentages of RTS depending on which moment the preoperative sports participation percentage was chosen. They found that 94 % of the patients were active in sports preoperatively ‘during life’, but only 36 % of the patients were still active in sports ‘at time of surgery’. Hence, the rates of RTS after TKA compared with these two different preoperative percentages were 36 and 81 %, respectively. Nine of the 13 TKA studies clearly defined the time period of scoring the preoperative sports participation. Argenson et al. [42] defined the preoperative sports moment as ‘at the time of surgery’ (RTS 86 %), Wylde et al. [53] used ‘3 years before surgery’ (RTS 73 %) and Lefevre et al. [43] and Huch et al. [44] used ‘participation during life’ (RTS 63 and 36 %, respectively) as the preoperative sports moment.

Seven of the nine studies reported the overall percentages of patients who RTS after UKA. Mean percentages of RTS varied from 74 % to more than 100 %, meaning that more patients participated in sports postoperatively than preoperatively. Four of these seven studies clearly described that the time period for the preoperative sports participation level was at the ‘presymptomatic phase’ with described RTS percentages of 93, 95, 98 and 75 % [14, 47, 51, 53].

### Pooling of Data of Sports Participation, RTS and Time to RTS

#### Pre- and Post-Operative Sports Participation and RTS

Data of ten TKA studies could be pooled (Table 4). Preoperatively, 1436 patients performed some type of low-impact sports a total of 1265 times. These sports included walking, swimming, golf and cycling (mean of 0.9 sports per patient), while 202 patients participated in an intermediate-impact type of sports, such as hiking, mountain climbing and downhill skiing (mean of 0.1 sports per patient), and 107 took part in a high-impact type of sport (mean of 0.1 sports per patient), such as running, tennis and ball sports.

**Table 4:** Pooled data of pre- and postoperative sports participation, RTS and time to RTS

Impact	Sports participation preoperative			Sports participation postoperative			Time to RTS		
	No. of sports	No. of patients	Average no. of sports/patient	No. of sports	No. of patients	Average no. of sports/patient	Time (weeks)	No. of patients	Average time (weeks)
TKA	(*n* = 7 studies)			(*n* = 10 studies)			(*n* = 4 studies)		
Low	1265	1436	0.9	1262	1524	0.8	4682.5	370	13
Intermediate	202	1436	0.1	132	1524	0.1	105.6	9	12
High	107	1436	0.1	51	1524	0.03	232.5	9	26
Total	1574	1436	1.1	1445	1524	1.0	5020.6	388	13
UKA	(*n* = 6 studies)			(*n* = 7 studies)			(*n* = 2 studies)		
Low	612	509	1.2	629	562	1.1	2680.2	222	12
Intermediate	237	509	0.5	155	562	0.6	280.6	18	16
High	91	509	0.2	33	562	0.1	30	3	10
Total	940	509	1.9	817	562	1.5	2990.8	243	12

*RTS* return to sports, *TKA* total knee arthroplasty, *UKA* unicondylar knee arthroplasty

In total, these 1436 patients practised preoperatively an average of 1.1 sports per patient, of which 80 % were low impact, 13 % were intermediate impact and 7 % were high impact. Postoperatively, 1524 patients performed some type of low-impact sports 1262 times (mean of 0.8 sports per patient), 132 a type of intermediate-impact sport (mean of 0.09 sports per patient) and 51 a type of high-impact sport (mean of 0.03 sports per patient). In total, these 1524 patients practised postoperatively an average of 0.95 sports per patient, of which 87 % were low impact, 9 % intermediate impact and 4 % high impact. RTS after pooling resulted in 94 % of patients returning to low-impact sports, 64 % to intermediate-impact sports and 43 % to high-impact sports. Two included studies for pooling were rated as having a low risk of bias [44, 50]. Pooled data from these two studies resulted in 337 sports practised by 549 patients (mean of 0.6 sports per patient: 58 % low impact, 25 % intermediate impact and 17 % high impact) preoperatively. Postoperatively, these 549 patients practised 155 sports (mean of 0.3 sports per patient), of which 85 % were low impact, 8 % intermediate impact and 7 % high impact. Two of the pooled studies used a similar definition of the time of the assessment of the preoperative sports level, namely ‘during life’ [43, 44]. Pooling of these data resulted in 398 patients performing a total of 209 sports preoperatively (mean of 0.5 sports per patient: 50 % low impact, 39 % intermediate impact and 11 % high impact). Postoperatively, 396 patients performed 93 sports (mean of 0.2 sports per patient), of which 79 % were low impact, 13 % were intermediate impact and 8 % high impact. All pooled data, specified by impact of sports, are summarised in Table 4.

Data of seven UKA studies were pooled (Table 4). Preoperatively, 509 patients practised some type of low-impact sport 612 times (mean of 1.2 sports per patient), an intermediate-impact sport 237 times (mean of 0.5 sports per patient) and a high-impact sport 91 times (mean of 0.2 sports per patient). In total, these 509 patients practised preoperatively an average of 1.9 sports per patient, of which 70 % were low impact, 22 % intermediate impact and 8 % high impact. Postoperatively, 562 patients performed some type of low-impact sports 629 times (mean of 1.1 sports per patient), an intermediate-impact sport 155 times (mean of 0.6 sports per patient) and a high-impact sport 33 times (mean of 0.1 sports per patient).

In total, these 562 patients practised postoperatively an average of 1.5 sports per patient, of which 77 % were of low impact, 19 % of intermediate impact and 4 % of high impact. RTS after pooling resulted in 93 % of patients returning to low-impact sports, >100 % of patients returning to intermediate-impact sports and 35 % to high-impact sports. There was only one article with a low risk of bias which could be included for pooling of data [14]. In this study, 83 patients practised 381 sports preoperatively (mean of 4.6 sports per patient: 49 % low impact, 36 % intermediate impact and 15 % high impact). Postoperatively, 238 sports were still being practised by these 83 patients (mean of 2.87 sports per patient), of which 64 % were low impact, 32 % intermediate impact and 4 % high impact. Three of the pooled studies used a similar definition of the time of the assessment of the preoperative sports level, namely ‘before the onset of any restricting knee symptoms’ [14, 47, 51]. Pooling of these data resulted in 194 patients performing a total of 563 sports preoperatively (mean of 2.9 sports per patient: 56 % low impact, 31 % intermediate impact and 13 % high impact).

Postoperatively, these 194 patients performed 415 sports (mean of 2.1 sports per patient), of which 71 % were of low impact, 26 % of intermediate impact and 3 % of high impact.

#### Time to RTS

Four articles considered time to RTS after TKA. Argenson et al. [42] reported a mean time of 6 months and Hopper and Leach [52] a mean time of 4.1 months to return to mainly low-impact sports. Bock et al. [41] reported an overall mean time of 4.7 months to return to both low- and higher-impact sports, and Lefevre et al. [43] reported on time to return to one specific high-impact type of sport, namely judo, with a mean of 5.2 months in former black belt judokas. Pooling of the time to RTS data (Table 4), 388 patients needed an average time of 13 weeks after TKA to RTS, of which 95 % concerned low-impact sports. The average time for nine patients to return to intermediate-impact sports was 12 weeks and for nine patients it took an average of 26 weeks to return to high-impact sports. None of these included studies scored a low risk of bias.

Three studies contained data regarding overall time to RTS after UKA. Hopper and Leach [52] reported an overall mean time to RTS of 3.6 months to return to low-impact sports. Naal et al. [14] and Walker et al. [47] described an overall RTS after UKA within 3 months of 46 and 56 %, respectively, and within 6 months of 69 and 78 %, respectively.

The last two authors studied return to both low- and higher-impact sports. By pooling the data of 243 patients (Table 4), a mean time of 12 weeks to RTS after UKA was found, of which 91 % concerned low-impact sports. The average time for 222 patients to return to low-impact sports was 12 weeks, for 18 patients to return to intermediate-impact sports this was 16 weeks, and it took an average of 10 weeks for three patients to return to high-impact sports. None of these studies included for pooling data scored a low risk of bias.

### Physical Activity

Regarding specific outcome measures of PA after TKA, UCLA scores were retrieved from three studies. Chang et al. [49] described a mean UCLA score of 4.5/10 (4.5 from a maximum possible score of 10) preoperatively and 4.8/10 postoperatively, and 9 % had a score higher than 6/10. Keeney et al. [55] described pre- and postoperative scores of patients younger than 55 years of 3.4/10 and 4.6 /10, respectively, and of patients between 65 and 75 years of 3.8/10 and 4.9/10. Bock et al. [41] described only a mean postoperative score of 5.9/10 in active patients. The Tegner-Lysholm score was described twice; it was 1.3/10 preoperatively and 3.5/10 postoperatively, with 24 % of scores higher than 5/10 in the study by Diduch et al. [54] and postoperatively a score of 3.9/10 was described in the study by Bock et al. [41]. The Grimby score was described only postoperatively in two studies; twice with scores of 2.8/6 [38, 40].

Regarding specific outcome measures concerning PA after UKA, two studies retrieved UCLA scores. Fisher et al. [51] scored a mean score of 4.2/10 before surgery and a score of 6.5/10 after surgery. Walker et al. [47] measured a mean score of 5.3/10 preoperatively and 6.7/10 postoperatively, with two-thirds of the scores >7. Walker et al. [47] also scored a Tegner of 2.9/10 preoperatively and 3.5/10 postoperatively. A Grimby score was measured in only one article, which showed a score of 3.9/6 postoperatively [38]. These PA scores show that after TKA, patients can regularly return to mild-to-moderate activities and UKA patients can return to moderate-to-high activities.

### Rehabilitation and Confounding Factors

Eight of the 18 included studies described information about the rehabilitation protocol followed after KA, typically not much more than mentioning that ‘full weight bearing was allowed’ or ‘all patients underwent standardised rehabilitation’ (not otherwise specified). Naal et al. [14] and Lo Presti et al. [48] gave the best descriptions by saying that patients were advised not to RTS before a sufficient muscular recovery of both quadriceps and hamstrings was reached.

Whether confounders were taken into account concerning RTS after KA was scored separately in our data extraction form (Tables 1 and 2). Five of the 18 studies adjusted for confounding: Bradbury et al. (50) found negative influences of restricting co-morbidities and complications, and positive influences of motivation and preoperative sports level on RTS, the latter confirmed by Naal et al. [14].

Age was mentioned as a possible confounder in eight studies, but only in five of these studies was this confounder adequately adjusted for; in four studies age did not have any influence on RTS and only Naal et al. [14] reported a negative influence of older age on RTS after UKA. Chatterji et al. [40], Huch et al. [44], Keeney et al. [55] and Wylde et al. [53] found an influence of sex—men were more able to RTS than women—but Naal et al. [14] did not find any influence of sex. A negative influence of high body weight on RTS was described in four studies. Three studies [44, 47, 53] listed specific patient-reported reasons for restricted sports participation after KA. Discouragement from their surgeons, mainly to high-impact types of sports, was one reason, in addition to pain, functional problems, instability and loss of motivation or loss of confidence. Moreover, the importance of counselling advice from the surgeon was mentioned in six studies, in four of which it was explicitly stated that patients were advised not to resume high-impact sports after KA. Only in the article by Lefevre et al. [43], concerning the judokas, did the influence of this advice seem low because many patients returned to sports despite the surgeon’s recommendations to the contrary. This therefore suggests a positive influence of motivation on RTS.

## Discussion

### Main Results

Patients are able to return to both low- and higher-impact sports after both TKA and UKA, with overall percentages varying from 36 to 89 and from 74 to >100 %, respectively. Participation in sports seems more likely after UKA than TKA, with mean total numbers of sports postoperatively of 1.1–4.6 sports per patient after UKA and 0.2–1.0 after TKA. RTS after TKA for low-impact sports was 94, 64 % for intermediate-impact sports, and 43 % for high-impact sports. For UKA, these numbers are 93, >100 and 35 %, respectively. These findings were confirmed by the PA scores of patients, which are higher after UKA than after TKA, namely return to mild-to-moderate activities after TKA and return to moderate-to-high activities after UKA. Time to RTS took 13 weeks after TKA and 12 weeks after UKA, with 95 and 91 %, respectively, concerning low-impact sports.

### Limitations and Strengths

A common limitation to all systematic reviews, including ours, is that some papers were overlooked. To overcome this problem, we performed an extensive search with sensitive search criteria and synonyms, and by making use of the expertise of a clinical librarian. Another limitation of this systematic review was that it consists of studies with broad heterogeneity in investigated study populations, defined baseline characteristics, chosen outcome measures and, of course, in research quality. Although systematic reviews with meta-analysis are generally seen as ‘a high quality of evidence’, we believe that given these limitations, our findings are at most of moderate quality. According to the outcome measures, many self-designed sports questionnaires were used. This kind of research is prone to so-called ‘recall bias’, as many rely on the patient’s ability to describe their sporting activities of several years previously. Moreover, different PA outcome measures were described, which were mostly not validated. The UCLA scale was most commonly used. Although the intrinsic disadvantage of the UCLA is that it is a categorical measure, it is a validated scale and until 2009 it seemed to be the most appropriate scale available for assessing PA levels in patients undergoing joint arthroplasty [56].

With respect to confounding factors, it is notable that in only seven of the 18 included studies was there a clear definition of the time of assessment of the preoperative sports level given. Considering the definitions used, such as ‘at time of surgery’, ‘at presymptomatic phase’ or ‘during life’, this has a significant effect on the reported RTS percentage, as Huch et al. [44] have also clearly shown. Other confounding factors that should have been adjusted for in determining percentages of RTS are sex, BMI, restricting comorbidities, complications, and psychosocial factors such as motivation and kinesiophobia of the patients. Conflicting results of a possible negative influence of age on postoperative activity have been mentioned previously, but the influence of age on RTS was also not clear from our included studies. Only a few included studies adequately adjusted for some or all of these confounding factors, resulting in an assessment of moderate or high risk of bias in 15 of 18 studies. In five of 13 included TKA studies and in four of eight included UKA studies, possible influences are stated concerning advice given by the surgeon that should also be taken into account. It is reasonable to assume that negative recommendations from their surgeons concerning high-impact types of sports will negatively influence a patient’s return to (especially) higher-impact sports, even if the patient had had the intention of doing so. Furthermore, the percentage of RTS is dependent on the preoperative sports level and (sports) rehabilitation.

A strength of the present study is that it provides a systematic overview of the literature concerning RTS and time to RTS after KA, including PA-specific outcome measures, while differentiating between TKA and UKA and pooling all extracted data. For this purpose, we selected only articles containing data of both pre- and postoperative sports participation, time to RTS and/or specific PA measurements. Most other reviews included general knee function scores like OKS (Oxford Knee Score) and the WOMAC (The Western Ontario and McMaster Universities Osteoarthritis Index) tool, which are generally accepted Patient Reported Outcome Measures (PROMs) nowadays. However, recently it has been stated that using these instruments has substantial disadvantages for the assessment of knee function with respect to activity and participation [57].

### Comparison with the Medical Literature

In 1996, Vail and Mallon [36] stated that published information on sports participation after joint arthroplasty is retrospective, limited in scope and primarily anecdotal in origin.

Almost 20 years later, negative advice concerning high-impact activities after joint arthroplasty is still more speculative than evidence-based. Concerning these sports recommendations, the general consensus is that return to low-to-intermediate-impact sports within 3–6 months is possible without any problems, while high-impact sports should be discouraged and high-contact athletic activities should be avoided [17, 20, 58, 59]. In contrast, the article by Lefevre et al. [43] showed that 63 % of former black belt judokas resumed their high impact sport, and Mont et al. [60] conducted a promising study with high-level tennis players, all of whom were also able to resume their sport after TKA. Although long-term effects of high-impact sports on outcomes of TKA need to be determined, both studies proved that return to high-impact sport is actually possible. The discussion includes risks of instability, periprosthetic fractures, bearing surface wear, early aseptic loosening, and subsequently premature revisions after high-impact sports. If one considers this subject from a purely mechanical point of view, it seems apparent that the bearing surface wear rate is directly related to the cycles of use. However, accumulating data suggest that prosthetic wear is not simply a function of time in situ, but rather a function of use [61]. During activities such as hiking or jogging, between 40 and 60 degrees of knee flexion high joint loads of 5–10 times body weight can occur, something that not all knee designs are capable of absorbing, so high polyethylene inlay stress may occur [62]. While some studies indeed found higher radiological wear and potential implant failure in active patients, they did not show an increase in revision rates due to high activities at mid-term [63]. This means that the feared higher risk for survival reductions after TKA in active patients cannot be confirmed. However, length of follow-up is not yet adequate to be able to make definitive conclusions on this matter [64]. On the other hand, recent advances in implant technology, surgical techniques and prosthetic designs and materials, and survival rates of new and improved types of KAs are promising for patients with high demands [65]. Several systematic reviews have concluded that patients and orthopaedic surgeons do not necessarily worry about the same things after joint replacement surgery, that patients should be encouraged to become active after joint replacement, and that further research in this area should be stimulated [16, 66–70].

### Clinical Implications

While younger and more active patients who undergo joint replacement may have higher expectations regarding activity, the literature suggests that nowadays they actually do not participate in functional levels of sports so often after knee replacement [20]. For example, Kersten et al. described that almost half of TKA patients did not meet health-enhancing PA guidelines and they were less active as a normative group [71]. After performing our review, the question arises: Is it due to a lack of will on the part of the patients that they are not always active after TKA? Or are they also highly influenced by negative advice from their orthopaedic surgeons regarding return to sports, as well as other possible restricting factors? Due to the fact that fulfilment of patient expectations after KA is considered to be a predictive criterion of satisfaction, the value of exploring a patient’s expectations regarding activities after knee replacement has been proven [72].

Historically, participating in sports after joint replacement has been discouraged. Although evidence on this subject is still sparse and of low quality, we can learn from this review that these negative recommendations are still not evidence-based and that actually it is possible to play many different sports after knee replacement surgery. Since postoperative outcomes and return to preoperative sports activity levels are influenced by many factors, individual characteristics, preoperative lifestyle, sport levels, motivation and patient preferences should be taken into account when one considers recommendations for athletic activity after joint replacement [73]. To optimise results, patients who demand higher levels of activity should be carefully selected. Since it seems that more patients can RTS and also to higher-impact types of sports after UKA in comparison with TKA, the choice of type of implant should also be considered. For individuals with limited anteromedial or only lateral compartment concerning types of OA, ‘as limited prosthetic constraint as possible’ and ‘as much retention of a “normal knee feeling” as possible’ are desirable.

Papalia et al. [70] recently found comparable RTS activity rates in patients undergoing TKA and UKA, but they based their conclusion on only one article. Regarding the results of our extensive systematic review, we, like Boyd et al. [74], tend to recommend a UKA over a TKA when indicated for a patient who wishes to remain highly active in a sport.

### Recommendations

Based on this review concerning RTS after KA, we strongly recommend using the same language concerning generalising a clear definition of preoperative sports level in future studies. It seems most rational to define the (real and only) preoperative sports level as the ‘presymptomatic phase’ and not the moment ‘at time of surgery’. In other words, preoperative sports level should be based on the phase when the patient was not yet restricted in participating in his or her preferred sports because of osteoarthritic knee complaints.

There is still no real reliable and valid method to analyse PA levels, although the level of activity seems an important prognostic factor, as well as a valuable outcome measure in the assessment of orthopaedic disorders [75]. PROMs have gained importance in both clinical practice and medical research and not only to patients and clinicians, but also to regulators, policy makers and health insurance authorities. But skewing and ceiling effects of currently used PROMs have been described, so using these would not be sufficient for reporting PA outcomes after KA [76]. So-called performance-based outcome measures (PBMs) are defined as assessor-observed measures of tasks classified as ‘activities’ using the International Classification of Functioning, Disability and Health (ICF) model of the World Health Organisation (WHO). PBMs are strongly related to patient self-efficacy in actual performance of function, and have been suggested as possible complementary objective measurement tools next to existing PROMs [77]. In predicting return to work in musculoskeletal diseases, PBMs were shown to strengthen the prognostic value of self-reporting modestly, from 9 to 16 % [78, 79]. The measurement of physical function is complex since it contains multi-dimensional constructs. After performing a systematic review, Dobson et al. [80] recommend further good quality research in investigating the measurement properties of PBMs in people with hip and/or knee OA. Following this conclusion, we would like to recommend investigating the possible added value of PBMs to currently used PROMs in predicting RTS after KA.

A lack of evidence is also apparent with regard to the rehabilitation of highly functioning individuals and those who wish to RTS after knee replacement, but there are some promising results that support a more aggressive rehabilitative approach [81]. Remarkably, hardly any information concerning rehabilitation could be extracted from the studies included in our review, while this seems to be a significant issue. We agree that muscular rehabilitation is important and Healy et al. [67] stated that stretching and strengthening programmes could enhance athletic performance after knee replacement, which could actually prevent injuries and protect joint reconstructions. We recommend performing more research on the (possibly protective) role of a more extensive rehabilitation after KA.

In the absence of consensus from the literature with respect to long-term survival rates especially after performing high-impact sports, there is a need for good quality prospective trials. From our review, it can be concluded that for some patients, some types of high-impact sports are possible after KA.

In the meantime, the ‘intelligent participation’ recommendations of Kuster et al. [37] should be considered. They do not only look at the impact of the sport on the joints, but also take into account prior experiences and the way a patient will perform his or her sport. If activities such as skiing, hiking or tennis were not to be performed as a regular endurance activity but on a recreational basis only, they would be less harmful. Moreover, when, for example, shortcuts and steep descents are avoided during hiking, walking slowly downhill and using ski poles can reduce knee joint loads by 20 %. It would also be acceptable for skilled skiers to ski on flatter slopes, avoiding hard snow conditions and moguls, for 1–2 weeks per year. However, it would seem unwise to start such technically demanding sports activities after knee replacement, due to higher joint loads in unskilled performers and because of the high risk of injuries such as periprosthetic fractures.

## Conclusion

Our systematic review showed that return to sports and physical activity is possible after both TKA and UKA, with percentages varying from 36 % to more than 100 %. Participation in sports seems more likely—including to higher-impact types—after UKA than after TKA, although after both surgeries patients tend to return to lower-impact types of sports. Time to RTS took 13 weeks after TKA and 12 weeks after UKA, respectively, with low-impact sports making up more than 90 % of cases. However, overall study quality of the included studies was limited due to confounding factors being insufficiently taken into account in most studies.

## Electronic supplementary material

Supplementary material 1: Appendix S1. Search strategy (DOCX 80 kb)

Supplementary material 2: Appendix S2. Criteria list for assessment of risk of bias (DOCX 109 kb)

Supplementary material 3: Appendix S3. Levels of impact on knee joint of different types of sports participation^a^ (DOCX 62 kb)
