# Supplementary material for: Return to Sports and Physical Activity After Total and Unicondylar Knee Arthroplasty: A Systematic Review and Meta-Analysis
Source: Sports Med. 2016 Jan 7;46:269–92. doi: 10.1007/s40279-015-0421-9 (PMC4728176; doi:10.1007/s40279-015-0421-9)
Supplement: Supplementary file 1 — Supplementary material 1: Appendix S1. Search strategy (DOCX 80 kb) [file 40279_2015_421_MOESM1_ESM.docx]

## Electronic Supplementary Material Appendix S1. Search strategy

PubMed:
("Arthroplasty, Replacement, Knee"[MeSH] OR "Knee Prosthesis"[MeSH] OR knee arthroplast*[tiab] OR knee replacement*[tiab] OR Knee Prosthes*[tiab]) AND ("Sports"[MeSH] OR "Motor Activity"[MeSH] OR "Athletes"[MeSH] OR sport*[tiab] OR physical*[tiab] OR athlet*[tiab] OR recreat*[tiab]) AND ("Recovery of Function"[MeSH Terms] OR return[tiab] OR recover*[tiab])

Embase:
Database(s): Embase Classic+Embase 1947 to 2014 March 25 Search Strategy:
# Searches Results
1 exp knee arthroplasty
2 exp knee prosthesis
3 (knee arthroplast* or knee replacement* or Knee Prosthes*).ti,ab,kw.
4 1 or 2 or 3
5 exp sport
6 exp physical activity
7 athlete
8 (sport* or physical* or athlet* or recreat*).ti,ab,kw.
9 5 or 6 or 7 or 8
10 convalescence
11 (return or recover*).ti,ab,kw.
12 10 or 11
13 4 and 9 and 12

SPORTDiscus:
( DE "TOTAL knee replacement" OR ( DE "KNEE" AND DE "ARTHROPLASTY" ) OR knee arthroplast* OR knee replacement* OR knee prosthes* ) AND ( ( DE "SPORTS" OR DE "SPORTS events" OR DE "PHYSICAL activity" ) OR ( sport* OR physical* OR athlet* OR recreat* ) ) AND ( return OR recover* )
