# Supplementary material for: Return to Sports and Physical Activity After Total and Unicondylar Knee Arthroplasty: A Systematic Review and Meta-Analysis
Source: Sports Med. 2016 Jan 7;46:269–92. doi: 10.1007/s40279-015-0421-9 (PMC4728176; doi:10.1007/s40279-015-0421-9)
Supplement: Supplementary file 2 — Supplementary material 2: Appendix S2. Criteria list for assessment of risk of bias (DOCX 109 kb) [file 40279_2015_421_MOESM2_ESM.docx]

## Electronic Supplementary Material Appendix S2. Criteria list for assessment of risk of bias

1. **Study participation**

- Source population/population of interest is adequately described; low = fully mentioned, moderate = partially mentioned, high = not mentioned
- Clear description of power analysis; low = mentioned, high = not mentioned
- Recruitment period and place of recruitment of patients; low = fully mentioned, moderate = partially mentioned, high = not mentioned
- Description of inclusion and exclusion criteria; low = fully mentioned, moderate = partially mentioned, high = not mentioned
- Clear description of baseline characteristics: age, body mass index, comorbidities and preoperative sports level/outcome measure; low = fully mentioned, moderate = partially mentioned, high = not mentioned

1. **Study attrition, follow-up**

- Follow-up of at least 12 months; low = > 12 months, high = < 12 months
- Adequate response rate % loss to follow-up; low = < 20%, high = > 20%
- Information about non-responders versus responders: age, body mass index, comorbidities and preoperative sports level; low = fully mentioned, moderate = partially mentioned, high = not mentioned

1. **Prognostic factor measurement**

- Clearly described information about the surgery performed: type of prosthesis used: UKA (medial or lateral) or TKA; low =mentioned, high = not mentioned
- Specified description of implant used (company, fixation method); low = fully mentioned, moderate = partially mentioned, high = not mentioned

1. **Outcome measurement**

- Clear definition of outcomes with special attention to definition of preoperative sports level, pre- and postoperative sports participation, return to sports (level and impact thereof), time to return to sports, satisfaction about activities and/or specific outcome measures for activity; low = mentioned, moderate = partly mentioned, high = not mentioned
- Valid and reliable measurement of outcomes are used, including blinding of outcome assessors; low = mentioned, moderate = partly mentioned high = not mentioned
- Same method and setting of outcome measurement for all study participants; low = fully mentioned and no large spreading of moments of outcome measurement; moderate = partially mentioned and/or large spreading of follow-up; high = not mentioned or different

1. **Study confounding**

- Important confounders mentioned: at least obesity (body mass index > 30), restricting comorbidities and described preoperative sports level (presymptomatic phase or just before surgery phase) and other possible reasons for restriction of sports participation (like pain, precaution, negative sports advice, no motivation regarding return to sports, or surgical complications); low = taken into account, moderate = partially mentioned, high = not taken into account at all
- Information about rehabilitation protocol used provided; low = fully described, moderate = partially described, high = no description
- Method used for missing data if > 20 % loss of follow-up; low = taken into account, high = not taken into account at all

1. **Analysis and reporting**

- Clear presentation of analysis and significance of primary outcomes; low = fully mentioned, moderate = partially mentioned, high = not mentioned
- Authors reported use of one or more methods to reduce bias (standardisation, matching, adjustment in multivariate model, stratification, propensity scoring), assessed dose–response in some way (subgroup, regression) or justified sample size; low = fully mentioned, moderate = partially mentioned, high = not mentioned
- Reporting of all results, no selective reporting; low = no selective reporting, moderate = probably selective reporting, high = clearly selective reporting
